# Exploration of microRNAs and their targets engaging in the resistance interaction between wheat and stripe rust

**DOI:** 10.3389/fpls.2015.00469

**Published:** 2015-06-30

**Authors:** Hao Feng, Bing Wang, Qiong Zhang, Yanping Fu, Lili Huang, Xiaojie Wang, Zhensheng Kang

**Affiliations:** ^1^State Key Laboratory of Crop Stress Biology for Arid Areas and College of Plant Protection, Northwest A&F UniversityYangling, China; ^2^College of Life Sciences, Northwest A&F UniversityYangling, China

**Keywords:** plant resistance, degradome sequencing, microRNA, *Puccinia striiformis* f. sp. *tritici*, wheat

## Abstract

Wheat stripe rust, caused by *Puccinia striiformis* f. sp. *tritici* (*Pst*), is one of the most destructive diseases of wheat worldwide. miRNAs are important regulators, they play very central roles in plant organ development, vegetable phase change and defense responses. In this study, two miRNA libraries from wheat cultivar Xingzi 9104 (XZ) challenged with the avirulent *Pst* race CYR32 and sterile water were constructed, respectively. A total of 596 miRNA candidates were obtained. 420 wheat-specific candidate miRNAs were screened in adult plants challenged with *Pst* using microarray-based analyses. We analyzed the abundance of candidate miRNAs, and the levels of a subset of candidate miRNAs were determined by quantitative real time PCR (qRT-PCR). The qRT-PCR results indicated that some miRNAs were involved in the incompatible interaction between wheat and *Pst*. In addition, we identified some miRNAs differentially expressed in different leaves. Additionally, the target genes of wheat miRNAs were confirmed by using degradome sequencing technology. Most of the annotated target genes are related to signal transduction, energy metabolism, and other functions. We selected some target genes for relative expression analysis using qRT-PCR, and found that RabGAP/TBC domain-containing protein, zinc finger protein and Cysteine-rich receptor-like protein kinase 41 may play important role in the incompatible interaction between XZ and CYR32. Intriguingly, miRNAs and target gene seem to form a complicated regulation network that regulates the wheat-*Pst* interaction. Our data provide the foundation for evaluating the important regulatory roles of miRNAs in the wheat-*Pst* interaction.

## Introduction

MicroRNAs (miRNAs) are endogenous non-coding small RNAs, typically of 21–24 nucleotides (nt) in length, that play important regulatory roles by repressing gene translation or degrading target mRNAs in eukaryotic organisms at the post-transcriptional levels (Bartel, 2004). In plants, mature miRNAs are processed by the RNase III enzyme Dicer-like1 (DCL1) from single-stranded RNA precursors capable of forming imperfectly complementary hairpin structures. miRNAs are loaded into the RNA-induced silencing complex (RISC) to regulate the expression of corresponding target genes (Kurihara et al., 2006; Cuperus et al., 2010). Since 2002, when the first plant miRNAs were discovered in *Arabidopsis*, miRNAs have been shown to play important roles in diverse plant processes, such as organ development, phase change, signal transduction, and biogenesis regulation (Llave et al., 2002; Reinhart et al., 2002).

Plant miRNAs are important regulators and also play very important roles in plant defense responses (Yu et al., 2012). Pathogens are considered to be one of the most serious factors threatening plant growth and production. To reduce pathogen damage, plants have evolved many adaptive response mechanisms to improve their tolerance and resistance capacity, including gene regulation mediated by miRNAs. In Arabidopsis, miR393 was the first miRNA implicated in pathogen-associated molecular pattern (PAMP)-triggered immunity (PTI), which was specifically induced by a *Pseudomonas syringae* infection and contributes to resistance by repressing auxin signaling (Navarro et al., 2006).

Wheat stripe rust is caused by *Puccinia striiformis* f. sp. *tritici* (*Pst*), and is one of the most destructive wheat diseases in the world. Stripe rust often causes a yield loss from 10 to 70% in susceptible varieties. Breeding and the rational utilization of disease-resistant varieties has been shown to be the safest, most effective, most economical and most environmentally sound method for controlling wheat stripe rust (Chen, 2005; Dodds and Rathjen, 2010). Therefore, understanding the interactions between wheat and *Pst* is important for developing strategies to improve disease resistance. Whether miRNAs are involved in the mechanism of wheat resistance against *Pst* is still a major open question. It is therefore of high interest to identify wheat miRNAs involved in the resistance interaction between wheat and *Pst*.

Wheat has a huge, 17-gigabase-pair, hexaploid genome. It is rather complex, and 80% of the genome consists of highly repetitive sequences (Brenchley et al., 2012; Mayer et al., 2014). This problem resulted in a delay of wheat miRNA research relative to model plants, such as *Arabidopsis* and rice. Yao et al. (2007) used 454 sequencing technology to successfully clone 58 miRNAs from wheat for the first time. With the development of new technologies (e.g., Solexa), a powerful tool for analyzing miRNA abundance has become available (Szittya et al., 2008; Breakfield et al., 2012). This approach has highlighted the benefits of providing a more thorough qualitative and quantitative description of gene expression. Using this technology, Xin et al. (2010) reported that wheat miRNAs were responsive to powdery mildew infection, revealing that miRNAs could be involved in the interaction between plants and pathogenic fungi. miRNAs are non-coding RNAs, so their target genes need to be identified first to better understand the role(s) of miRNAs in detail. Previous miRNA studies on the target identification were only predicted by using bioinformatic analyses (Yao et al., 2007; Xin et al., 2010). Meanwhile, 5′ RACE technology was another popular method for verifying miRNA target genes (Lin et al., 2010; Li et al., 2011a,b). The main limitation of this approach is that predicted target genes must be independently validated. During the past few years, high-throughput degradome sequencing has been established to address this problem (Addo-Quaye et al., 2008; Li et al., 2010; Zhou et al., 2010). These new technologies also provide the opportunity to explore the detailed roles of miRNAs in the interaction between wheat and its pathogens.

To examine the differential accumulation of miRNAs that are responsive to *Pst*, the present study surveyed miRNA populations in XZ using high-throughput sequencing technology after a challenge with *Pst*. In addition, the corresponding target genes were identified using degradome sequencing. Identifying miRNAs and their targets will lay a comprehensive foundation for unraveling the complex miRNA-mediated regulatory network in the XZ-*Pst* interaction.

## Materials and methods

### Plant materials and treatments

Wheat cultivar “Xingzi 9104” and stripe rust race CYR32 were obtained from the Institute of Plant Pathology, Northwest A&F University, Yangling, Shaanxi, China. After a 4-week vernalization of germinated XZ seeds, they were sown in pots and placed in a growth chamber under the following conditions: 16 ± 2°C with supplemental light for 16 h per day (240 μmol/m^2^/s photon flux density) and water as needed. Four weeks later, the seedlings were shifted to 25 ± 2°C. After the plants grew to the boot stage, wheat flag leaf surfaces were inoculated with CYR32 urediospores (AT-I) and sterile water (AT-M) as described (Feng et al., 2011). Plant material was collected at 0, 24, 48, and 120 h post inoculation (hpi), immediately frozen in liquid nitrogen and stored at −80°C for library construction.

### Construction of small RNA libraries and sequencing

Total RNA of each sample was extracted using Trizol Reagent (Invitrogen, Carlsbad, CA) according to the manufacturer's instructions. Total RNA extracted from each sample was treated with DNAse I for 30 min at 37°C to remove any contaminating genomic DNA. The total RNA quantity and purity were analyzed of Bioanalyzer 2100 and RNA 6000 Nano LabChip Kit (Agilent, CA, USA) with RIN number >7.0. Two small RNA libraries (AT-M and AT-I) were constructed using the corresponding RNA pools, which were mixed with the same amount of RNA samples from 24, 48, and, 120 hpi. Approximately 1 μ g of total RNA were used to prepare small RNA library according to protocol of TruSeq Small RNA Sample Prep Kits (Illumina, San Diego, USA). And then we performed the single-end sequencing (36 bp) on an Illumina Hiseq2500 at Huada Gene Research Institute (Shenzhen, China) following the vendor's recommended protocol. Two biological replicates were used for each library construction.

### Analysis of sRNA sequencing data

Data processing followed the procedures provided by LC Sciences Service at the LC-BIO (Hangzhou, China), which was suitable for hairpin prediction of plant miRNAs. Briefly, the raw reads were subjected to the Illumina pipeline filter (Solexa 0.3), and then the dataset was further processed with an in-house program, ACGT101-miR (LC Sciences, Houston, Texas, USA) to remove adapter dimers, junk, low complexity, common RNA families (rRNA, tRNA, snRNA, snoRNA) and repeats based on the GenBank (http://www.ncbi.nlm.nih.gov/genbank) and Rfam (http://rfam.sanger.ac.uk/) databases. Subsequently, unique sequences with length in 18–25 nucleotide were mapped to specific species precursors in miRBase 20.0 (http://microrna.sanger.ac.uk) by BLAST search to identify known miRNAs and novel 3p- and 5p- derived miRNAs. Length variation at both 3p- and 5p- ends and one mismatch inside of the sequence were allowed in the alignment. The unique sequences mapping to specific species mature miRNAs in hairpin arms were identified as known miRNAs. The unique sequences mapping to the other arm of known specific species precursor hairpin opposite to the annotated mature miRNA-containing arm were considered to be novel 5p- or 3p- derived miRNA candidates. The remaining sequences were mapped to other selected species precursors (with the exclusion of specific species) in miRBase 20.0 by BLAST search, and the mapped pre-miRNAs were further BLASTed against the specific species genomes to determine their genomic locations. The unmapped sequences were BLASTed against the wheat genome database (ftp://ftp.plantgdb.org/download/Genomes/TaGDB/TAbac175.bz2) and mRNA database (ftp://ftp.plantgdb.org/download/Genomes/TaGDB/TAest175.bz2), and the hairpin RNA structures containing sequences were predicated from the flank 120 nt sequences using RNAfold software (http://rna.tbi.univie.ac.at/cgi-bin/RNAfold.cgi) and analyzed by MIREAP (http://sourceforge.net/projects/mireap/). The criteria for secondary structure prediction were: (1) number of nucleotides in one bulge in stem (≤12) (2) number of base pairs in the stem region of the predicted hairpin (≥16) (3) cutoff of free energy (kCal/mol ≤ 15) (4) length of hairpin (up and down stems + terminal loop ≥ 50) (5) length of hairpin loop (≤200) (6) number of nucleotides in one bulge in mature region (≤4) (7) number of biased errors in one bulge in mature region (≤2) (8) number of biased bulges in mature region (≤2) (9) number of errors in mature region (≤4) (10) number of base pairs in the mature region of the predicted hairpin (≥12) (11) percent of mature in stem (≥80).

### High-throughput sequencing abundance profile analysis

The high-throughput sequencing abundance profile analysis was based on the sequence read number of each library. The first step was to normalize miRNA sequence reads in the *Pst*-inoculated wheat plants and control plants to tags per million. Calculation of the *P*-value to compare miRNA expression between the different groups (AT-M and AT-I) was based on previously established methods (Audic and Claverie, 1997). Specifically, the log_2_ ratio equals log_2_ (miRNA AT-I/miRNA AT-M). The *P*-value formula was as follows:

(1) $$\begin{array}{l} p(x|y)={\left(\frac{N_{2}}{N_{1}}\right)}^{y}\frac{(x+y)!}{x!y!{\left(1+\frac{N_{2}}{N_{1}}\right)}^{\left(x\;+y\;+\;1\right)}}\\ \;C(y\leq y_{\min}|x)=\sum_{y\;=\;0}^{y\;\leq \;y_{\min}}p(y|x)\\ \;D(y\geq y_{\max}|x)=\sum_{y\;\geq y_{\max}}^{\infty }p(y|x), \end{array}$$

where *N_1_* is the total read number of all miRNAs in the control library, *N_2_* is the total number of reads of all miRNAs in the *Pst* inoculation library, *x* is the number of reads for an miRNA in the control library, and *y* is the number of reads for an miRNA in the *Pst* inoculation library. All calculations were performed on the BGI Bio-Cloud Computing platform. miRNA tags per million of <1 were filtered from both libraries.

### Construction of wheat degradome libraries and sequence analysis

Target genes of both known miRNAs and new miRNAs were verified by wheat mRNA degradome sequencing following the published parallel analysis of RNA Ends (PARE) protocol (German et al., 2009). Total RNA samples from the AT-I used for small RNA sequencing library construction were also used for miRNA target identification. Two biological replicates were used for library construction. The purified cDNA library was used for cluster generation on Illumina's Cluster Station and then sequenced on Illumina GSIIx following the manufacturer's instructions. Raw sequencing reads were obtained using Illumina's Pipeline v1.5 software following sequencing image analysis by the Pipeline Firecrest Module and base-calling by the Pipeline Bustard Module. Extracted reads were analyzed with the software package CleaveLand 3.0 and blasted with wheat transcripts generated from the same sample. The annotation of candidate target genes was performed using the Blast2GO Gene Ontology Functional Annotation Suite (GO) and the Kyoto Encyclopedia of Genes and Genomes (KEGG).

### Primer design, cDNA synthesis and qRT-PCR

The concentration of pure RNA was measured using a NanoDrop 1000 spectrophotometer (Thermo Fisher Scientific, USA). Reverse transcription of mature miRNAs and candidate target genes was performed as described (Feng et al., 2011, 2012). cDNA samples were diluted 20-fold with sterile water before being used as the template in qRT-PCR analyses. Quantitative PCR amplifications were performed with a CFX96 Real-Time System (Bio-Rad) with SYBR Green I chemistry (Invitrogen). The translation elongation factor 1 alpha-subunit (EF) gene (GenBank accession no. M90077) was used as a control (Kong and Yang, 2010). PCR conditions were taken from Feng et al. (2011, 2012). Relative levels of miRNAs in *Pst*-inoculated plants were calculated as the fold change vs. mock-inoculated plants at that time point using the comparative 2^−ΔΔ^CT method. Three biological replicates were performed for each experiment. All primers are listed in Supplemental Table 1.

### miRNA array and RNA gel blot analysis

The miRNA array platform was designed according to Jia et al. (2010). A total of 420 miRNAs were selected for a microarray-based screen. Antisense probes for each miRNA (20 μM) were printed in duplicate on a Hybond-N+ membrane using the Genetix Qpix2 robot (Supplemental Table 2). The array platform included three external controls (MAC1, MAC2, and MAC3).

RNA samples extracted from the first leaves of seedlings and flag leaves of adult plants were used for hybridization. Total RNA was extracted with Trizol™ Reagent (Invitrogen). A 100 μg aliquot of total RNA was separated on a 15% denaturing PAGE gel, and small RNAs (14–28 nt) were extracted from the gel and used for the array. Small RNAs were dephosphorylated with Antarctic phosphatase (New England Biolabs) and radiolabeled with γ−^32^P-ATP and T4 phosphonucleotide kinase (PNK). Radiolabeled small RNAs were hybridized to the miRNA array at 37°C for 12 h in hybridization buffer (50% formamide, 5x SSPE, and 5x Denhardt's solution). The membrane was washed three times (20 min each) with washing buffer (2x SSC, 0.1% SDS) at 42°C. Following hybridization, detection was performed using Phosphor-Imager screens (FLA-7000, Fujifilm). Two biological replicates were used for each experiment.

For the RNA gel blot assay, 80 μg of total RNA were isolated from the first leaves of seedlings and from the second top leaves and flag leaves of adult plants and separated on a denaturing 15% urea-PAGE gel before being electro-transferred on to Hybond-N^+^ membrane using a Semi-Dry Transfer Cell (Bio-Rad). DNA oligonucleotide probes were labeled with γ−^32^P-ATP and PNK. Hybridization was carried out as described above. All the probes are listed in Supplemental Table 1.

## Results

### An overview of the sRNA sequencing results

To identify novel miRNAs in wheat, two small RNA libraries (AT-I and AT-M) were sequenced. Deep sequencing the libraries generated 10,543,243 and 13,859,814 raw reads for AT-I and AT-M plants, respectively (see Supplemental Figure 1A). After removing low-quality and junk sequences (reads < 15 nt), a total of 10,329,636 and 13,586,483 mappable reads were analyzed for the length distribution of small RNAs (15–32 nt long) (see Supplemental Figure 1B). The 24-nt mRNAs were found to have the highest read abundance (see Supplemental Figure 2). Additionally, mappable reads were compared with known wheat mRNAs using the Rfam and Repbase databases. After discarding 2,596,235 and 4,043,428 reads, and aside from 5,166,240 and 5,613,765 no-hit sequences, remaining 2,547,161 and 3,909,290 sequences were used to identify conserved miRNA homologs using miRBase. The set of sequences with perfect matches to the wheat genome was used to identify novel miRNAs in wheat. In this study, the nucleotide bias position analysis showed that the first nucleotide of the new wheat miRNA candidates generally tended to be (U) (see Supplemental Figure 3). We also analyzed the copy number of novel miRNAs in wheat (see Supplemental Figure 4). Most miRNAs had only one copy, comprising approximately 93% of total miRNAs; 33 miRNAs had two copies. PC-488 had the largest number of copies, with seven in our sequence database. Using the bioinformatics statistical results, we found that the number of wheat specific miRNAs was much higher than the number of conserved miRNAs.

### Identification of known miRNAs from wheat

First, we identified known miRNAs (including conserved and non-conserved) by mapping unique sRNAs to wheat miRNAs in miRBase with fewer than four mismatches. In wheat, there are 44 wheat pre-miRNAs corresponding to 42 unique mature miRNAs, according to miRBase. In this study, 18 conserved miRNAs belonging to 11 families were identified, together with 30 non-conserved miRNAs (see Supplemental Table 2). Among these miRNAs, five novel conserved miRNAs and 17 novel non-conserved miRNAs were found. Six mature miRNAs (tae-miR1118-p5, tae-miR1121-p3, tae-miR1122-p5, tae-miR1123-p3, tae-miR1128-p5, and tae-miR1133-p5) were found in this study, whose precursors were recorded in miRBase with no original mature miRNA. At the same time, 15 -5p or -3p new mature miRNAs, for which related miRNAs originating from the opposite arms of the same pre-miRNAs have been reported in miRbase, were detected. The detection of these miRNAs illustrates the sensitivity of the high-throughput sequencing approach in miRNA discovery.

### Identifying novel miRNAs from wheat

**Figure 1:**
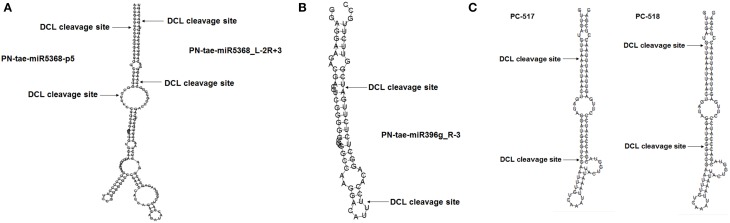
**Typical secondary structures of miRNAs. (A)** Two distinct miRNAs (PN-tae-miR5368-p5 and PN-tae-miR5368_L-2R+3) originating from different arms of the same precursor. **(B)** Structure of a new tae-miR396 family member. **(C)** Two miRNAs (PC-517 and PC-518) are derived from the same arm of the same precursor.

In addition to detecting known miRNAs, the high-throughput sequencing data also made it possible to discover new miRNAs. As described above, we first mapped the unique sRNAs to the miRNAs of selected plants in miRBase. Eight candidates could be mapped to the selected plant miRNAs, the corresponding pre-miRNAs of which could potentially form hairpins and could also be mapped to the *Triticum aestivum* genome (see Supplemental Table 3). We identified five novel conserved miRNAs originating from five families and three novel non-conserved miRNAs. Interestingly, we identified two mature miRNAs originating from the same precursor, designated as PN-tae-miR5368_L-2R+3 and PN-tae-miR5368-p5 (Figure 1A). We also detected a new member of a conserved miRNA family in wheat, namely PN-tae-miR396g (Figure 1B). In Supplemental Table 4, we list miRNA candidates with extended sequences at the mapped genome positions that could potentially form hairpins. There are 14 miRNA candidates highly similar to other plant miRNAs. However, the read abundance of all candidates was weak, with the exception of PN-tae-miR530-5p_L+1.

There was still a large number of sRNAs un-mapped to selected miRNAs in miRBase. We mapped the results to the wheat genome database with no nucleotide mismatches. In total, 526 unique sequences, the extended sequences of which could potentially form hairpins, were identified as potential novel wheat miRNAs, which were deemed wheat-specific miRNA candidates (see Supplemental Table 5). We also identified many mature miRNA candidates that could be generated from distinct arms of the same precursor. PC-517 and PC-518, for example, are two special miRNA candidates derived from the same arm of a single precursor (Figure 1C). This may result from the differential recognition of cleavage sites by DCL.

### Bioinformatic analysis and biological verification of wheat miRNA expression in response to *Pst*

**Figure 2:**
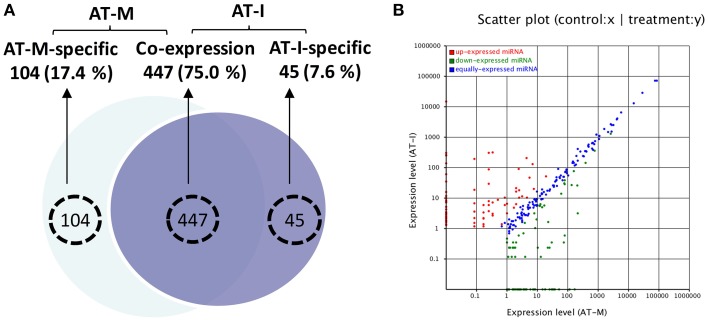
**Expression of miRNAs in wheat cultivar Xingzi 9104 in response to CYR32 at adult stage. (A)** Venn diagram of differentially expressed miRNA candidates in XZ at the adult stage in response to CYR32 (AT-I) and the mock control (AT-M). **(B)** miRNA scatter plot of the relative expression levels of miRNA candidates at adult stage XZ in response to CYR32 (y-achsis) and mock control (x-achsis).

To explore the function of miRNAs in the interaction between XZ and CYR32, we analyzed the AT-M and AT-I miRNA libraries to explore candidate miRNAs specifically induced by *Pst*. Out of 596 candidate miRNAs, 104 and 45 miRNA candidates were specifically expressed in AT-M or AT-I, respectively. The remaining 447 were expressed in both AT-M and AT-I (Figure 2A). We compared their relative expression in wheat challenged with *Pst* with the control. We found that miRNAs show various expression profiles in wheat challenged with *Pst* compared to the control (Figure 2B).

**Figure 3:**
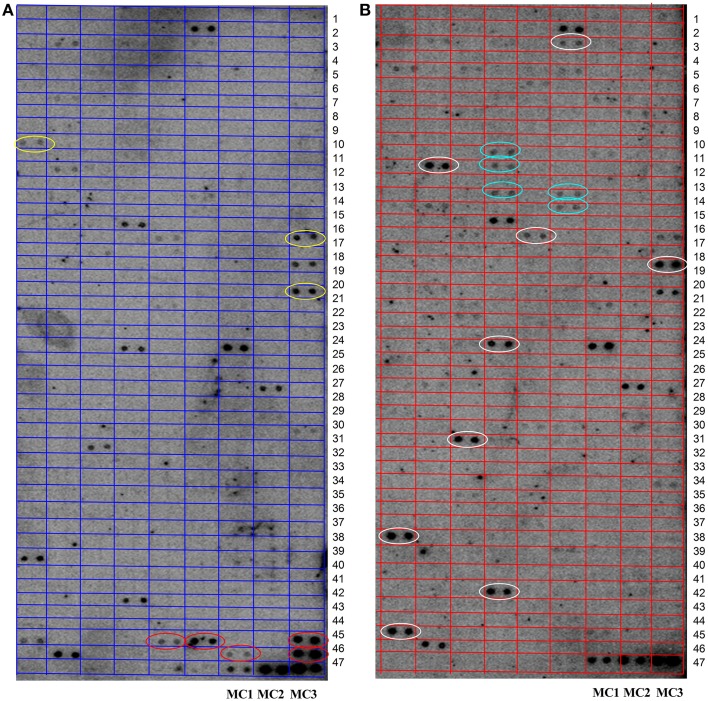
**Identification of novel miRNAs in response to Pst by microarray. (A)** RNA was extracted from the mock inoculated leaves (AT-M). **(B)** RNA was extracted from the leaves inoculated with Pst (AT-I). RNA samples from adult stages of XZ challenged with *Pst* together with control samples were used to screen novel miRNAs using array analysis. One hundred micrograms of total RNA was used for the array. The extracted small RNAs were 5′ end labeled and probed against the antisense DNA oligonucleotides spotted onto the membrane. MC1-3 were selected as external control to detect the feasibility of the system. White and yellow circles indicate miRNAs that are up- or down-regulated when leaves were challenged with *Pst*, respectively. Red and green circles highlight miRNAs expressed only in control leaves or only in test leaves, respectively.

According to the read number, a total of 420 miRNAs were selected for a microarray-based screen (see Supplemental Table 6). As shown in Figure 3, hybridization of external controls (MC1-3) was good, demonstrating that the hybridization system was working. Some miRNAs are stably expressed at both AT-M (A) and AT-I (B). Some miRNAs are expressed in both groups, however, with different expression levels. Compared with AT-M (A), eight miRNAs are up-regulated (white circles), and two miRNAs are down-regulated (yellow circles), when the leaves were inoculated with *Pst*. We also found five miRNAs specifically expressed in the control (red circles). Another five miRNAs are expressed solely when leaves were challenged with *Pst* (green circles).

### Validation of novel miRNAs specifically expressed during plant development

**Figure 4:**
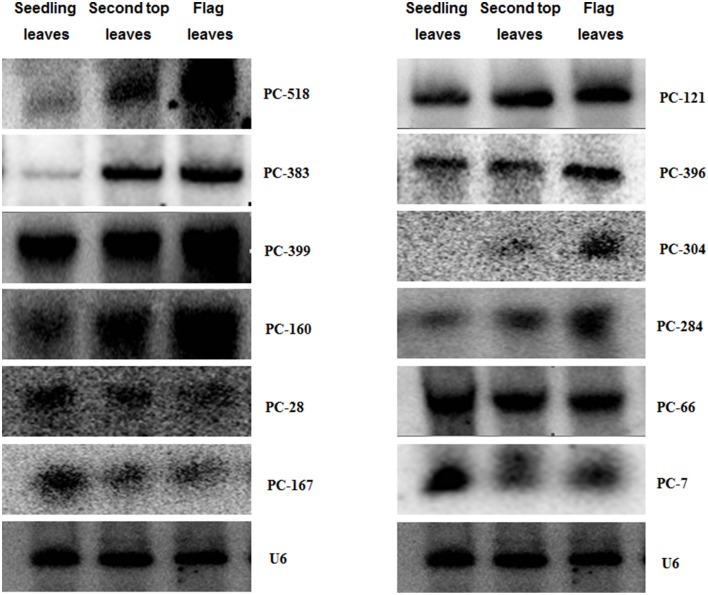
**Expression of miRNAs in wheat cultivar Xingzi 9104 at different growth stages**. Expression validation of miRNAs using an RNA gel blot. Total RNA (80 μg) extracted from the first leaves, second top leaves and flag leaves of XZ were used for RNA gel blot analysis. U6 was used as the loading control. The hybridization bands were approximately 21 bp.

In order to assess their potential role in developmental processes, we screened 26 candidate miRNAs in differently aged leaves samples of control plants through microarray. To confirm the miRNAs identified by this array, 12 miRNAs were further validated by RNA gel blot analysis. Their hybridization bands were approximately 21 bp (Figure 4), which represents the specific hybridization of mature miRNAs. The others 14 showed no signal or the wrong hybridization signal position (data not shown). In Figure 4, there were five total miRNAs (PC-518, PC-383, PC-160, PC-304, and PC-284) with a higher expression level at the adult stage, and three miRNAs (PC-28, PC-167, and PC-7) showing a higher expression level at the seedling stage. The others showed no obvious difference during these two growth stages. More importantly, the expression transition may be much earlier than the boot stage because the miRNA expression in the second top leaves had similar profiles to that in the flag leaves.

### Overview of the degradome sequencing results

**Figure 5:**
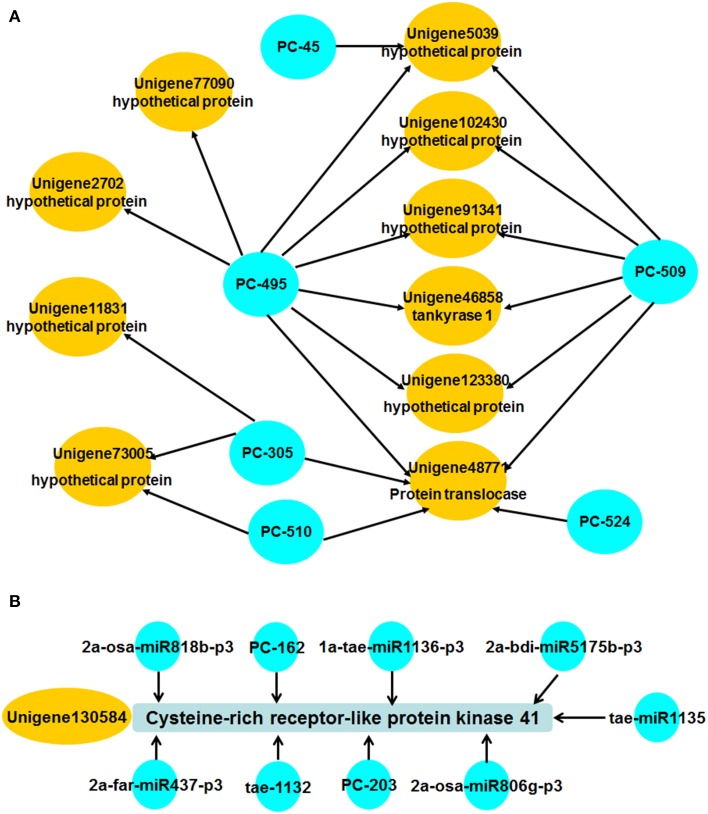
**Regulation network constructed with miRNAs and corresponding targets. (A)** Several miRNAs and their corresponding targets were selected to show the complex regulation net. One miRNA regulating several targets and one target being regulated by various miRNAs was the general phenomenon in this study. **(B)** Regulation network of unigene13,058 and the corresponding miRNAs.

In higher plants, most miRNAs function by cleaving their corresponding targets, and cleavage normally occurs at the tenth nucleotide of the complementary region between the miRNA and the mRNA (Bartel, 2004). We applied a recently developed high-throughput degradome sequencing technology that can identify miRNA targets at a global scale (Llave et al., 2002; Addo-Quaye et al., 2008; German et al., 2009). A library was constructed to identify miRNA target genes from wheat during *Pst* infection. We obtained 20,631,556 raw reads representing the 5′ ends of uncapped, poly-adenylated RNAs from the AT-I library (see Supplemental Table 7). After removing low-quality reads, redundancies, and duplications, a total of 20,069,266 unique raw reads were obtained. Because unigenes detected by degradome sequencing are incomplete (without the 5′ end), we mapped them to the transcriptome sequence data of XZ, and 5,016,864 signatures from AT-I library were perfectly mapped to the XZ transcriptome. Because of the lack of publicly available cDNA information on wheat, we used wheat cDNA information provided by the TIGR Wheat Genome Annotation Project. A total of 118,519 of these signatures were mapped to wheat-annotated cDNAs. The cleaved target transcripts were categorized into five classes according to the “height” of the degradome peak at each occupied transcript position. After mapping all miRNA candidates by using previously described methods (Addo-Quaye et al., 2008), there were 893 unigenes identified as target genes (see Supplemental Table 8). We found that there are still large numbers of miRNA targets that were not detected, so far. Only 219 miRNA targets were detected in this library. We also found that miRNA regulation in wheat is very complex. In AT-I, there were 34 miRNAs with only one target gene each, while the other miRNAs and their corresponding targets could construct a regulation network. Some miRNAs, such as PC-495, could even target eight different target genes. Additionally, unigene 48771 could be regulated by PC-495, PC-509, PC-510, PC-305, and PC-524 (Figure 5A). These findings indicate that many miRNAs and their targets are scattered across a very complex interaction network.

### Annotation of candidate target genes originating from wheat leaves

For the annotation, all the unigenes were aligned using BLASTX to the NCBI non-redundant protein database (nr) and the Swiss-Prot protein database using a cut-off *E*-value of 10^−5^ (see Supplemental Table 8). We found that most sequenced could not be annotated using known plant genes, and most unigenes with matches to the Swiss-Prot database could also be found in the nr database. Gene ontology (GO) enrichment analysis was performed to classify gene function for unigenes. The *E*-value distribution in AT-I library showed that the results were reliable (see Supplemental Figure 5A). Because the genome information for wheat is still not public, we found that most of the genes showed hits to *Hordeum vulgare, Brachypodium distachyon*, and *Oryza sativa* (see Supplemental Figure 5B), and the sequence similarity was very high, with most results reaching up to 80% (see Supplemental Figure 5C). These results indicate that the annotation was reliable.

Based on sequence homology, the candidate target gens can be categorized into several functional groups consisting of three domains, namely biological processes, cellular components and molecular function by using the GO annotation. By using KEGG, we found candidate targets involved in many important physiological and signal transduction pathways, including the TCA cycle, glycolysis, and gluconeogenesis, ascorbate metabolism, fatty acid metabolism, ubiquinone biosynthesis, oxidative phosphorylation, MAPK signaling, calcium signaling, and others.

Because XZ possessed resistance at adult stage, we aimed to find if there were several genes involved in stress response. Intriguingly, some key proteins involved in stress signal transduction were detected, such as protein kinase, transcription factors, and others (Table 1).

**Table 1:** **Typical targets of miRNA identified by degradome sequencing (*P* < 0.1)**.

**miRNA**	**Target**	**Alignment score**	**Alignment range**	**Cleavage site**	**Annotation**
tae-miR164	Unigene8858	4	270–290	281	WRKY2 transcription factor [*Triticum aestivum*]
2b-zma-miR169a_1ss21AG	Unigene14969	2	855–875	866	nuclear transcription factor Y subunit A-3 [*Zea mays*]
1a-tae-miR1433-p5	Unigene14969	4	853–875	866	nuclear transcription factor Y subunit A-3 [*Zea mays*]
PC-162	Unigene130584	3	123–146	137	Cysteine-rich receptor-like protein kinase 41 [*Triticum urartu*]
PC-190	Unigene118084	1.5	120–140	131	Peroxisome assembly protein 12 [*Aegilops tauschii*]
PC-277	Unigene148631	4	134–157	148	dehydrogenase [*Aequorivita capsosiphonis*]
PC-305	Unigene11831	3	628–651	642	disease resistance protein [*Aegilops tauschii*]
PC-328	Unigene53415	3	115–137	128	LRR receptor-like serine/threonine-protein kinase [*Aegilops tauschii*]
PC-375	Unigene85325	4	947–970	961	diacylglycerol kinase [*Arabidopsis thaliana*]
PC-377	Unigene36720	1	248–264	255	RNA-binding protein 39 [*Triticum urartu*]
PC-452	Unigene12315	3.5	99–122	113	3-hydroxyisobutyryl-CoA hydrolase [*Zea mays*]
PC-490	Unigene8446	1.5	888–908	899	TBC domain-containing protein [*Brachypodium distachyon*]

### Expression profiles of the candidate target genes and corresponding miRNAs during *Pst* infection

To confirm whether the candidate target genes were involved in the interactions between wheat and *Pst*, six unigenes involved in stress signal transduction were selected for transcript accumulation analysis in wheat after challenge with CYR32 at adult stage (AI24, AI48, and AI120), respectively (Table 2).

**Table 2:** **Relative transcription level of target genes and corresponding miRNAs in wheat challenged by *Pst***.

**Target Gene**	**AI24**	**AI48**	**AI120**	**Annotation**	**miRNA**	**AI24**	**AI48**	**AI120**
Unigene8858	1.58	1.32	0.98	WRKY2 transcription factor	tae-miR164	0.90	0.67	0.55
Unigene27318	90.36	2.34	8.50	Zinc finger protein	tae-miR1136-p3	1.08	0.50	0.64
Unigene130584	0.01	1.12	1.46	Cysteine-rich receptor-like protein kinase 41	PC-162	0.84	3.02	1.23
Unigene148631	0.96	1.54	2.37	Dehydrogenase	PC-277	0.52	0.74	0.46
Unigene53415	0.65	0.88	2.02	LRR receptor-like serine/threonine-protein kinase	PC-328	1.10	0.91	0.46
Unigene8446	43.83	0.79	0.99	TBC domain-containing protein	PC-490	0.91	0.96	0.43

*Six unigenes, identified as targets of miRNAs, together with the corresponding miRNAs, were selected for transcript accumulation analysis in wheat after challenge with CYR32 (AI24, AI48, and AI120), respectively. The data were normalized to the expression level of wheat translation elongation factor 1 alpha-subunit (EF). The relative expression level of them in the Pst-inoculated plants at each time point was calculated as the fold of the mock-inoculated plants at that time point using the comparative 2^-ΔΔ^ CT method. The experiments were repeated in triplicates of independent biological replicates using newly extracted RNA and synthesized cDNA samples*.

According to the results, we found these unigenes showed differential transcript accumulation. Importantly, unigene8446, unigene27318, and unigene130584 are dramatically induced/reduced in adult wheat responsing to *Pst*. We speculate they may play an important role in the incompatible interaction between XZ and CYR32. According to the GO analysis, they were annotated as RabGAP/TBC domain-containing protein, zinc finger protein and Cysteine-rich receptor-like protein kinase 41, respectively.

To further explore the interaction between miRNAs and target genes, we also detected the expression trends of the corresponding miRNAs according to the results of degradome sequencing with *p* < 0.1. We found most of them showed divergent expression trends between miRNAs and target genes. For example, both unigene8858 and tae-miR164 showed no significant induction when wheat was challenged with *Pst*. Unigene148631 and Unigene53415 were up-regulated at 120 hpi, while expression of the corresponding miRNAs PC-277 and PC-328 decreased at 120 hpi, respectively. While the expression of unigene8446, unigene27318, and unigene130584 were not consist with the corresponding miRNAs during *Pst* infection. Unigene8446 and unigene27318 were highly induced at 24 hpi with 43.83- and 90.36-folds compared with control, while PC-490 and tae-miR1136-p3 showed only two-folds down regulated expression at 48 and 120 hpi, respectively. The expression of unigene130584 was decreased with nearly a 100-folds, while the expression of PC-162 showed only 3.02-folds up regulation at 48 hpi. This may be caused by the various regulation of miRNAs. As mentioned above, the regulation network seems to be very complex. For example, unigene130584 could be regulated by nine miRNAs according to the result of degradome sequencing, although the *p*-value of most them was bigger than 0.1 (Figure 5B). Thus, the real interactions between miRNAs and their target should be bio-experimentally verified in further studies.

## Discussion

miRNAs are endogenous non-coding small RNAs that engage in regulatory functions at the post-transcriptional levels (Reinhart et al., 2002). Most miRNAs regulate the corresponding genes by directly cleaving target mRNAs during various plant biological progresses. Wheat cultivar XZ possesses resistance at adult stage to stripe rust, but the underlying reason for this defense was not clear. Histological and cytological aspects of adult XZ plant resistance to *Pst* were characterized in previous studies (Zhang et al., 2012). Additionally, many genes were shown to be induced by *Pst* during the adult stage but not during the seedling stage (Huang et al., 2013). miRNAs are important regulators, and they play very important roles in plant organ development, phase change and defense responses (Poethig, 1990; Aukerman and Sakai, 2003). Therefore, the identification of miRNAs and their targets will lay a comprehensive foundation for unraveling complex miRNA-mediated regulatory networks and their contribution in wheat responding to the *Pst* infection.

### Diverse wheat miRNAs

To follow up the hypothesis that miRNAs play a role in regulating wheat response to *Pst*, we constructed two miRNA libraries from XZ leaves by using the high-throughput Solexa sequencing approach. In this study, all the mappable reads were analyzed for the length distribution of small RNAs (15–32 nt long). The length distribution of miRNAs was consistent with the typical size range for Dicer-derived products (Sunkar and Zhu, 2004). The first nucleotide of the new wheat miRNA candidates generally tended to be (U). This finding is consistent with previous reports showing that miRNAs were loaded into RISC and assisted by AGO proteins, which have greater affinity with uracil in the 5′ terminus of miRNA, thus resulting in cloned miRNA sequences with a uracil nucleotide bias in the first position (Mi et al., 2008).

After bioinformatics analysis, we identified 56 common miRNAs (23 conserved and 33 non-conserved) and 540 wheat-specific miRNAs, which included results from leaves inoculated with *Pst* as well as control. This finding greatly increased the number of known wheat miRNAs. We screened the novel miRNAs and found distinct expression profiles at different growth stages of wheat. Many miRNAs are expressed in a tissue-specific or developmental stage-specific manner, thereby contributing to specific target gene expression (Inui et al., 2010). Many newly emergent non-conserved miRNAs are expressed in specialized tissue, or are present at relatively low abundances (Rajagopalan et al., 2006; Fahlgren et al., 2007). Temporal miRNA expression analysis in other plants also indicated that some miRNAs were expressed at only certain developmental stages (Aukerman and Sakai, 2003; Sunkar and Zhu, 2004; Rajagopalan et al., 2006; Zhang et al., 2011; Sun et al., 2014).

Recent deep sequencing of other plant small RNA libraries also demonstrated that plants express more non-conserved than conserved miRNAs (Rajagopalan et al., 2006; Fahlgren et al., 2007). Interestingly, many published miRNAs in miRBase were not detected in our study, but the miRNAs originating from the opposite strand were detected, and these have been referred to as miRNA^*^ in the past. In fact, miRNA^*^ have been shown to play an important role in some specific cases (Guo and Lu, 2010). In addition, our study revealed that the XZ wheat genome encoded more specific miRNA families than conserved miRNA families. We speculated that there was a mass of wheat miRNAs that had not been detected at different growth stages, and its regulatory network was very complex.

### Many specific miRNAs were expressed in response to *Pst*

The sequencing read number reflects the miRNA expression abundance at least to some extent. To define the contribution of miRNAs to resistance, we compared the sequencing read data from two separate miRNA libraries. In this study, most miRNAs were co-expressed in both AT-M and AT-I. Interestingly, nearly all the specifically expressed miRNAs showed a relative low read abundance. This observation could be explained by the hypothesis that highly expressed miRNAs are mainly involved in basic biological pathways, whereas less expressed miRNAs are responsible for regulating specific pathways (Glazov et al., 2008). In past few years, several studies demonstrated that wheat miRNAs were responsive to biotic and abiotic stresses. In 2010, 24 wheat miRNAs were identified as being responsive to powdery mildew infection in wheat via Solexa high-throughput sequencing (Xin et al., 2010). Gupta et al. (2012a) reported that miR167, miR171, miR444, miR1129, and miR1138 are players in resistance of wheat against rust. Followed that, Inal et al. (2014) presented that a number of the miRNAs such as miR2592s, miR869.1, miR169b were highly differentially regulated showing more than 200-fold change upon fungal-inoculation. And we also confirmed two conserved miRNAs and their corresponding target genes involved in the wheat-*Pst* interactions (Feng et al., 2013, 2014). Actually, most conserved miRNAs mentioned above were also identified in this study, and they were predicted to be *Pst* responsive miRNAs. In addition, we also sequenced lots of novel wheat miRNAs, most of which were not identified in other plants until now, all of these will lay a foundation for evaluating the roles of miRNAs in the XZ-CYR32 interaction comprehensively.

### Target identification and relative expression in response to *Pst*

To further explore the function of miRNA in defense against *Pst*, we identified the targets for wheat miRNAs using degradome sequencing. Some miRNAs were easily detectable, but we could not identify their targets. We propose that miRNAs are kept in reserve at a low level, and only increase and excert their role under specific conditions. In this study, nearly 600 novel miRNAs were detected in total, while only a portion of their targets was identified. Although almost all of the identified targets were not annotated, which may be attributed to incomplete genome sequences, we still identified many important genes response to stress, including transcription factors, protein kinases, resistance related genes, and so on.

According to a previous study, the main evidence for the resistance at the adult stages is the duration and strength of HR (Zhang et al., 2012). Actually, we isolated several significant target genes that regulated HR. In addition, we also identified many resistance/defense related genes, and the expression of them could be regulated by the *Pst*. The phenomenon showed that, there may be various pathways for XZ to regulate the resistance to *Pst*. Among them, we got three important target genes (RabGAP/TBC domain-containing protein, zinc finger protein and Cysteine-rich receptor-like protein kinase 41), which were not mentioned in previous studies for XZ resistance mechanisms (Huang et al., 2013). It was noteworthy that all of them reached the peak or nadir at 24 hpi, which was the key time point for the establishment of resistance interaction (Zhang et al., 2012). RabGAP/TBC domain-containing protein possessed Rab GTPase activator activity, and it could increase the rate of GTP hydrolysis by a GTPase of the Rab family (Itoh et al., 2006). In addition, it was confirmed that RabGAP/TBC domain-containing protein of grapevine were induced after beneficial microorganism *Trichoderma harzianum* T39 inoculation and contributed the host resistance (Palmieri et al., 2012). Zinc protein is a small protein structural motif, they were also confirmed to play an important role in plant resistance to pathogens (Gupta et al., 2012b). In this study, we found the expression of RabGAP/TBC domain-containing protein and zinc finger protein were induced vigorously, which indicated the important function in the resistance response of XZ to *Pst*. However, Cysteine-rich receptor kinase 41 was drastically down regulated when ZX was challenged by *Pst*. Cysteine-rich receptor-like kinase (CRK) belongs to the receptor-like kinase family. Until now, not much is known about this kind of genes in wheat. In 2013, a wheat CRK gene *TaCRK1* was isolated from *Rhizoctonia cerealis*-resistant wheat, and the down regulation of *TaCRK1* transcript did not obviously impair resistance to *R. cerealis* (Yang et al., 2014). In barley, the transcripts of a CRK gene *HvCRK1* were observed to accumulate transiently following *Bgh* inoculation of susceptible barley, and played negative effect on basal resistance against *Bgh* Rayapuram et al., 2012). However, several CRKs were transcriptionally induced by exposure to O_3_, which were very similar to responses to microbes or pathogen-associated molecular patterns (PAMPs) in *Arabidopsis* (Wrzaczek et al., 2010). This may be caused by the different regulation among genes between monocotyledons and dicotyledons. Combining the expression level and the related reports in other plants response to pathogens, we speculated these three genes may contribute to the resistance interaction positively or negatively. Future studies will enable us to decipher the molecular mechanisms of them, and the certain evidences will be found in next few years.

### MiRNA and protein coding genes construct an interaction network

In previous studies, it was found that most miRNA-complementary sites are located in the coding regions of the target, and they are found in the 3′ or 5′ UTRs in only a few cases (Carrington and Ambros, 2003; Baulcombe, 2004). In this study, we found most miRNA-complementary sites to be located in 3′ UTRs. This finding is consistent with the results of the Yao group (Yao et al., 2007). They found that out of all the miRNA-complementary sites for 16 novel wheat miRNA targets, 11 are within 3′ UTRs, only three are in a coding region, and two are in a 5′ UTR.

It is not known whether there is a one-to-one relationship between miRNAs and their targets. In previous reports, miRNAs were shown to regulate multiple members of the same gene family or genes related to a common biochemical pathway. For example, miRNA398 regulated the whole family of Cu/Zn SOD genes (Bouche, 2010). In our study, we found only a few miRNAs followed that rule; miRNAs and target genes made up a complex interaction network. In fact, recent findings revealed that the same miRNA can target different gene families that participate in entirely unrelated biochemical pathways, and one target gene could be regulated by distinct miRNAs (German et al., 2009; Li et al., 2010). For this reason, these results may be a starting point for further exploration of molecular mechanism of wheat resistance to *Pst*.

## Conclusions

In conclusion, Solexa sequencing provided an accurate and efficient approach for studying small RNAs in wheat. We discovered a large number of miRNAs not only during plant development but also during *Pst* infection. The target genes for these miRNAs were also identified. Many genes are related to stress responses, and they constructed a regulation net with the corresponding target genes. Among them, three important target genes (RabGAP/TBC domain-containing protein, zinc finger protein, and Cysteine-rich receptor-like protein kinase 41) were identified, combining the high/low expression level and the related reports in other plants response to pathogens, we speculated these three genes may contribute to the resistance interaction positively or negatively. These results provided the basis for future miRNA function analysis in wheat resistance to *Pst*.

### Conflict of interest statement

The authors declare that the research was conducted in the absence of any commercial or financial relationships that could be construed as a potential conflict of interest.

## Abbreviations

AT-I: adult stage-inoculation
AT-M: adult stage-mock
CYR: Chinese yellow rust
DCL: DICER-LIKE
hpi: hours post inoculation
HR: hypersensitive response
miRNAs: microRNAs
PC: potential candidate
*Pst*: *Puccinia striiformis* f. sp. *tritici*
PTI: pathogen-associated molecular pattern triggered immunity
qRT-PCR: quantitative real-time-PCR
RISC: RNA-induced silencing complex
XZ: wheat cultivar Xingzi 9104.
