# Supplementary material for: Exploration of microRNAs and their targets engaging in the resistance interaction between wheat and stripe rust
Source: Front Plant Sci. 2015 Jun 30;6:469. doi: 10.3389/fpls.2015.00469 (PMC4485317; doi:10.3389/fpls.2015.00469)
Supplement: Supplementary file 7 [file Table7.DOC]

Supplemental Table 7. Data summary of degradome library

|  | **Raw Reads** | **Unique Raw Reads** | **cDNA Mapped Reads** | **Total Number**  **of input cDNAs** | **Number of**  **coverd cDNAs** |
| --- | --- | --- | --- | --- | --- |
| AT-I | 20,631,556 | 20,069,266 | 5,016,864 | 157,689 | 118,519 |

One library was constructed using the degradome sequencing technology for miRNA targets identification. AT-I, XZ challenged with CYR32 at adult stage. The samples collected at 24 hpi, 48 hpi, and 120 hpi from AT-I were mixed together for RNA extraction and sequencing.
