# Supplementary material for: Exploration of microRNAs and their targets engaging in the resistance interaction between wheat and stripe rust
Source: Front Plant Sci. 2015 Jun 30;6:469. doi: 10.3389/fpls.2015.00469 (PMC4485317; doi:10.3389/fpls.2015.00469)
Supplement: Supplementary file 9 [file Image1.PDF]

Supplemental Figure 1. Overview of wheat high-throughput sequencing.

To identify novel miRNAs of wheat, two small RNA libraries (AT-I and AT-M) were sequenced. A, total number of small RNAs. B, mappable small RNAs after removal of low-quality and junk sequences.

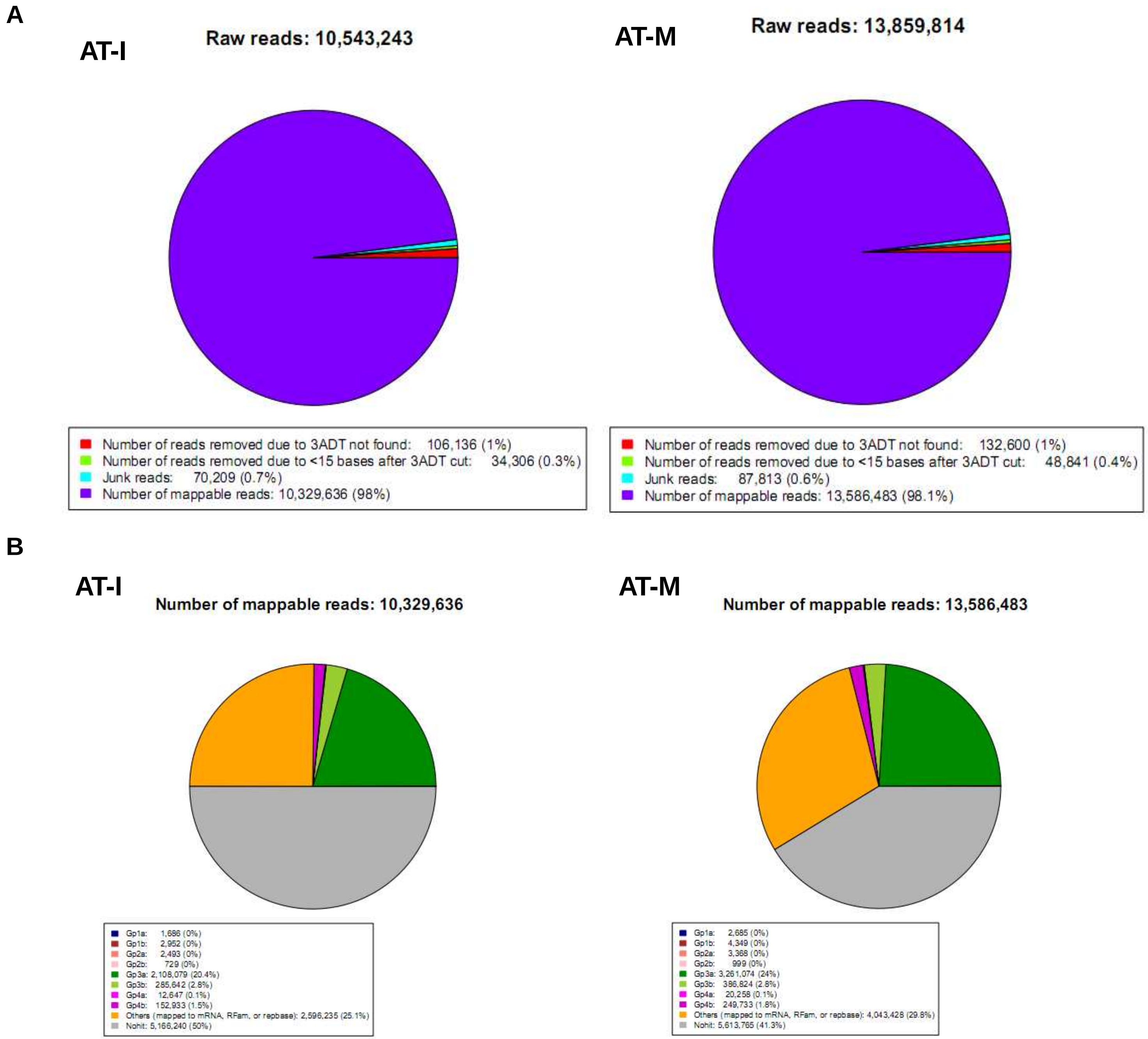
A
AT-I
Raw reads: 10,543,243
Number of reads removed due to 3ADT not found: 106,136 (1%)
Number of reads removed due to <15 bases after 3ADT cut: 34,306 (0.3%)
Junk reads: 70,209 (0.7%)
Number of mappable reads: 10,329,636 (98%)
AT-M
Raw reads: 13,859,814
Number of reads removed due to 3ADT not found: 132,600 (1%)
Number of reads removed due to <15 bases after 3ADT cut: 48,841 (0.4%)
Junk reads: 87,813 (0.6%)
Number of mappable reads: 13,586,483 (98.1%)
B
AT-I
Number of mappable reads: 10,329,636
Gp1a: 1,686 (0%)
Gp1b: 2,952 (0%)
Gp2a: 2,493 (0%)
Gp2b: 729 (0%)
Gp3a: 2,108,079 (20.4%)
Gp3b: 285,642 (2.8%)
Gp4a: 12,647 (0.1%)
Gp4b: 152,933 (1.5%)
Others (mapped to mRNA, RFam, or repbase): 2,596,235 (25.1%)
Nohit: 5,166,240 (50%)
AT-M
Number of mappable reads: 13,586,483
Gp1a: 2,685 (0%)
Gp1b: 4,349 (0%)
Gp2a: 3,368 (0%)
Gp2b: 999 (0%)
Gp3a: 3,261,074 (24%)
Gp3b: 386,824 (2.8%)
Gp4a: 20,258 (0.1%)
Gp4b: 249,733 (1.8%)
Others (mapped to mRNA, RFam, or repbase): 4,043,428 (29.8%)
Nohit: 5,613,765 (41.3%)
