# Supplementary figures and images for: Exploration of microRNAs and their targets engaging in the resistance interaction between wheat and stripe rust
Source: Front Plant Sci. 2015 Jun 30;6:469. doi: 10.3389/fpls.2015.00469 (PMC4485317; doi:10.3389/fpls.2015.00469)

Supplemental Figure 2. Length distribution of sRNAs.

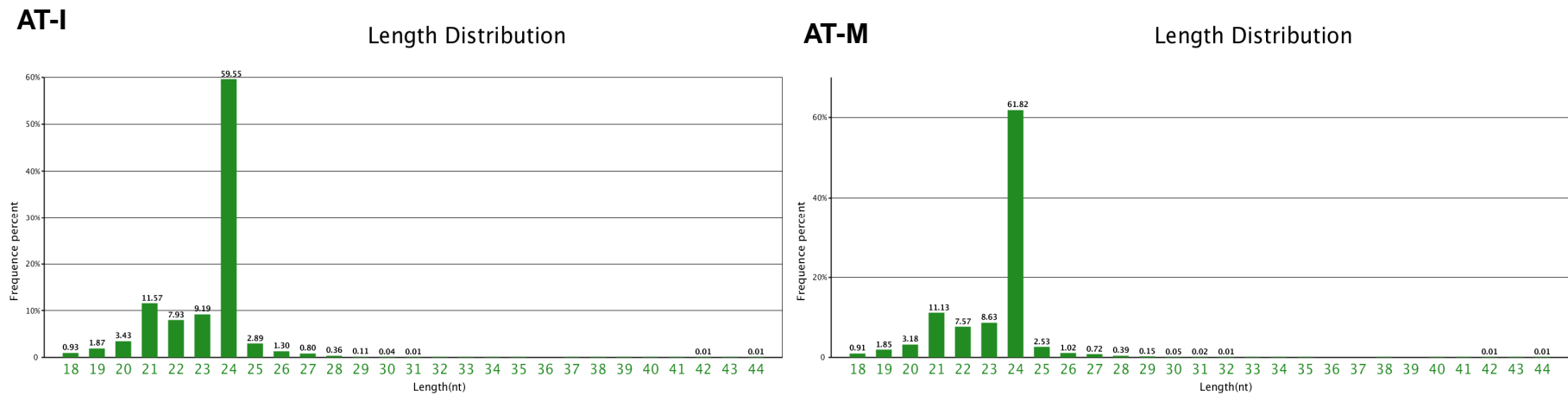

Supplement: Supplementary file 10 [file Image2.PDF]

**Supplemental Figure 4. Copy number of miRNAs.**

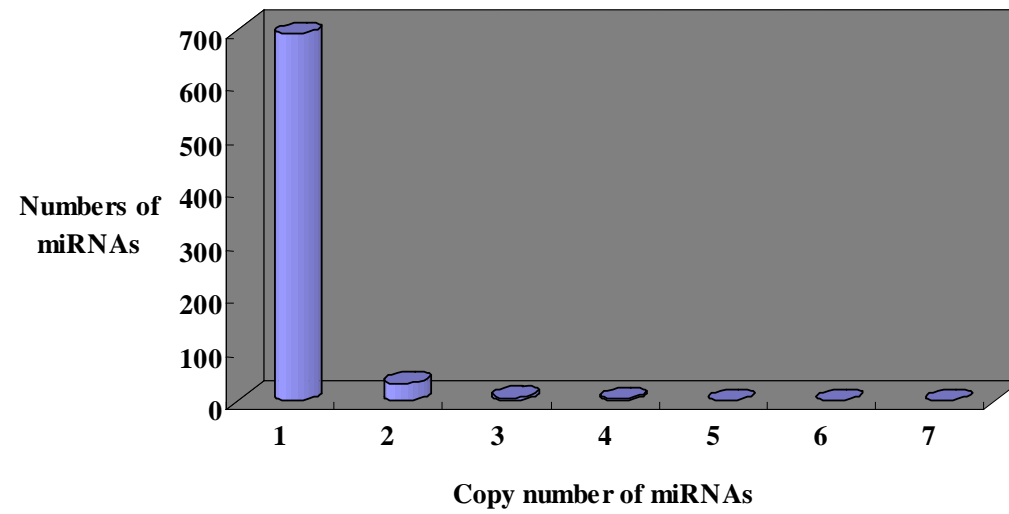

Supplement: Supplementary file 12 [file Image4.PDF]
