# Supplementary material for: Exploration of microRNAs and their targets engaging in the resistance interaction between wheat and stripe rust
Source: Front Plant Sci. 2015 Jun 30;6:469. doi: 10.3389/fpls.2015.00469 (PMC4485317; doi:10.3389/fpls.2015.00469)

Supplemental Figure 3. Nucleotide bias in each position of newly indentified miRNAs.

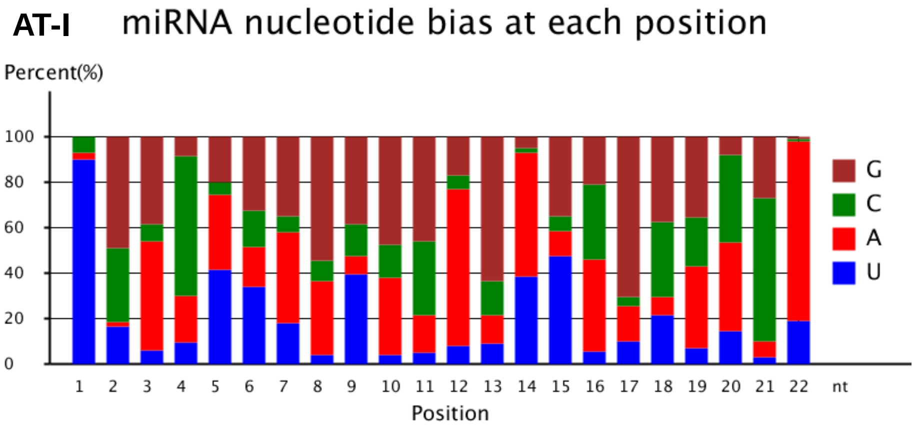

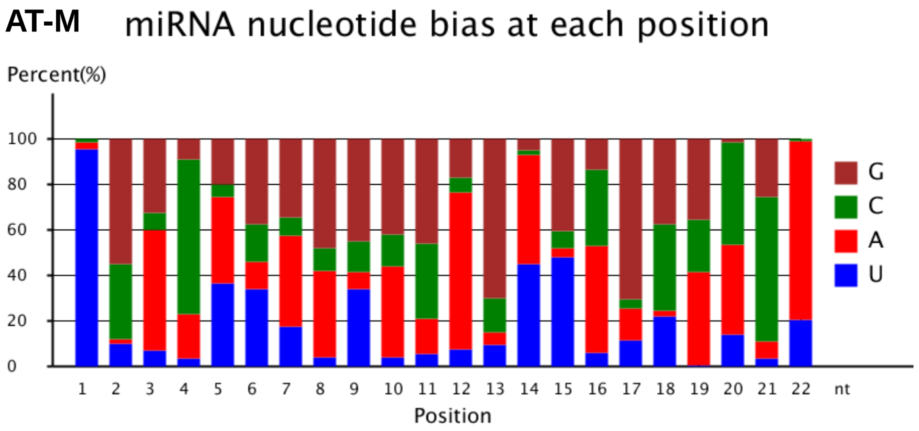

Supplement: Supplementary file 11 [file Image3.PDF]
