# Supplementary material for: Exploration of microRNAs and their targets engaging in the resistance interaction between wheat and stripe rust
Source: Front Plant Sci. 2015 Jun 30;6:469. doi: 10.3389/fpls.2015.00469 (PMC4485317; doi:10.3389/fpls.2015.00469)
Supplement: Supplementary file 13 [file Image5.PDF]

Supplemental Figure 5. Overview of unigenes classification using Gene ontology (GO) enrichment analysis

Target genes were verified using degradome sequencing technology. Samples were collected from XZ challenged with *Pst* at adult stage. Unigenes were annotated though Go ontology enrichment analysis. A, E-value distribution; B top-hit plant species distribution; C, the sequence similarity distribution.

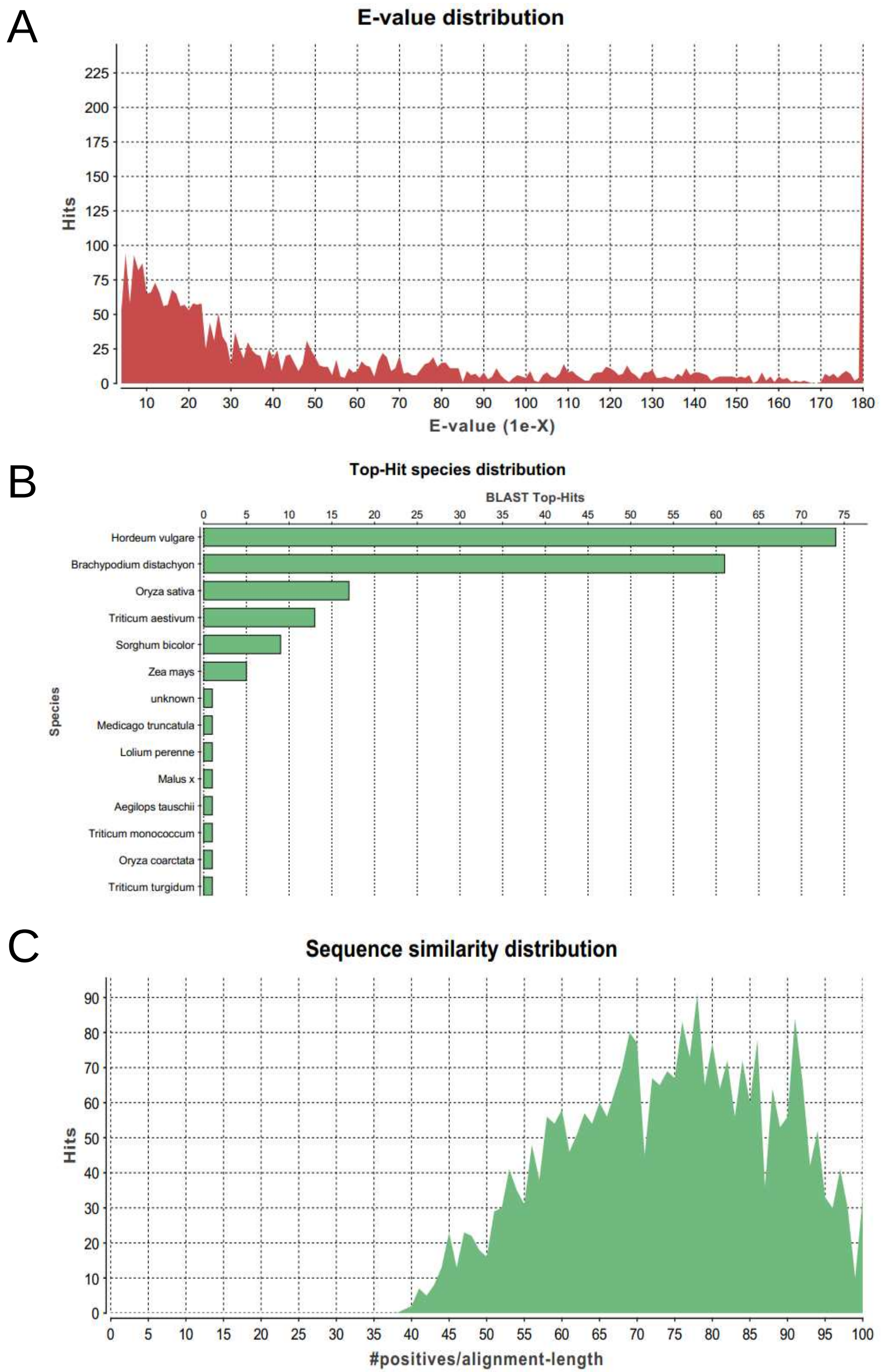
A
E-value distribution
Hits
E-value (1e-X)
B
Top-Hit species distribution
BLAST Top-Hits
Species
Hordeum vulgare
Brachypodium distachyon
Oryza sativa
Triticum aestivum
Sorghum bicolor
Zea mays
unknown
Medicago truncatula
Lolium perenne
Malus x
Aegilops tauschii
Triticum monococcum
Oryza coarctata
Triticum turgidum
C
Sequence similarity distribution
Hits
#positives/alignment-length
